# Supplementary material for: Hybrid coating prepared with PMMA/Ti-O-Si tested under vacuum conditions for use in nanosatellites
Source: PLoS One. 2025 Jan 9;20(1):e0313785. doi: 10.1371/journal.pone.0313785 (PMC11717194; doi:10.1371/journal.pone.0313785)
Supplement: Attachment — Submitted filename: This paper discusses a hybrid coating made of poly.docx [file pone.0313785.s002.docx]

This paper discusses a hybrid coating made of poly (methyl methacrylate) with SiO_2_-TiO_2_ particles (PMMA/SiO_2_-TiO_2_) developed for weight loss. I have some comments to modify the manuscript.

1. A paper has already been published in 2024 in which the researchers prepared the PMMA/SiO_2_-TiO_2_ composite. Is your synthetic protocol different from it? Please justify.

<https://doi.org/10.1016/S0169-4332(03)00865-1>

2. You can again check the reference style of the journal
3. How is the interaction between titanium oxide and silicon oxide identified in the Raman spectrum, and what is the wavenumber range for the Ti-O-Si bond?
4. What effects does the Raman spectrum's indication of the interaction between silicon oxide and titanium oxide have on the general characteristics and possible uses of the composite material?
5. The figures' quality is very poor. Kindly improve it. As you can see in the figure of SEM and figure 13, and 14, even the digits are not visible.
6. What is the novelty of your research paper? Kindly discuss it briefly in the conclusion section.

I suggest a major revision on the base of these comments.
