# Supplementary material for: Hybrid coating prepared with PMMA/Ti-O-Si tested under vacuum conditions for use in nanosatellites
Source: PLoS One. 2025 Jan 9;20(1):e0313785. doi: 10.1371/journal.pone.0313785 (PMC11717194; doi:10.1371/journal.pone.0313785)
Supplement: Attachment — Submitted filename: Response to Reviewers.docx [file pone.0313785.s003.docx]

**Editorial Board**

**PLOS ONE**

**Dear Komal Rizwan, PhD
Academic Editor**

According to your instructions, we have duly corrected our manuscript “**Hybrid coating prepared with PMMA/Ti-O-Si tested under vacuum conditions for use in nanosatellites”,** (**PONE-D-24-26920**) on the basis of the Referees` comments that you kindly sent to us. We have carefully addressed the reviewer’s recommendations and suggestions.

The changes made to the manuscript have been realized in the Microsoft Word file that we are submitting through the “Edit Revision” Internet access, in accordance al template of the journal. We have also answered each of the point raised by the Referees in the authors Responses to the Reviewers Comments file that we will be sending on line to Journal PLOS ONE.

All these recommendations have been marked in yellow color and the answers in blue color.

We hope that this version will be completely satisfactory now and would like to the referee for details revisions of the paper.

Sincerely,

**Dra. Genoveva Hernández-Padrón**

e-mail: [genoveva@unam.mx](mailto:genoveva@unam.mx)

**AUTHORS RESPONSES TO REVIEWERS COMMENTS**

Journal PLOS ONE **PONE-D-24-26920**

“HYBRID COATING PREPARED WITH PMMA/Ti-O-Si TESTED UNDER VACUUM CONDITIONS FOR USE IN NANOSATELLITES”

Here were refereeing to those comments raised by Reviewers 1 and 2 that are required to be answered by us, highlighted in blue. Additionally, we are writing all the changes that we introduced in the revised version of our manuscript, highlighted in yellow.

**Journal Requirements:**

1. When submitting your revision, we need you to address these additional requirements.

Please ensure that your manuscript meets PLOS ONE's style requirements, including those for file naming. The PLOS ONE style templates can be found at 
[https://journals.plos.org/plosone/s/file?id=wjVg/PLOSOne_formatting_sample_main_body.pdf](https://journals.plos.org/plosone/s/file?id=wjVg/PLOSOne_formatting_sample_main_body.pdf) and 
[https://journals.plos.org/plosone/s/file?id=ba62/PLOSOne_formatting_sample_title_authors_affiliations.pdf](https://journals.plos.org/plosone/s/file?id=ba62/PLOSOne_formatting_sample_title_authors_affiliations.pdf)

2. We note that your Data Availability Statement is currently as follows: All relevant data are within the manuscript and its Supporting Information files.

Please confirm at this time whether or not your submission contains all raw data required to replicate the results of your study. Authors must share the “minimal data set” for their submission. PLOS defines the minimal data set to consist of the data required to replicate all study findings reported in the article, as well as related metadata and methods ([https://journals.plos.org/plosone/s/data-availability#loc-minimal-data-set-definition](https://journals.plos.org/plosone/s/data-availability#loc-minimal-data-set-definition)).

For example, authors should submit the following data:

- The values behind the means, standard deviations and other measures reported;
- The values used to build graphs;
- The points extracted from images for analysis.

Authors do not need to submit their entire data set if only a portion of the data was used in the reported study.

If your submission does not contain these data, please either upload them as Supporting Information files or deposit them to a stable, public repository and provide us with the relevant URLs, DOIs, or accession numbers. For a list of recommended repositories, please see [https://journals.plos.org/plosone/s/recommended-repositories](https://journals.plos.org/plosone/s/recommended-repositories).

If there are ethical or legal restrictions on sharing a de-identified data set, please explain them in detail (e.g., data contain potentially sensitive information, data are owned by a third-party organization, etc.) and who has imposed them (e.g., an ethics committee). Please also provide contact information for a data access committee, ethics committee, or other institutional body to which data requests may be sent. If data are owned by a third party, please indicate how others may request data access.

**Reviewer #1**

This paper discusses a hybrid coating made of poly (methyl methacrylate) with SiO_2_-TiO_2_ particles (PMMA/SiO_2_-TiO_2_) developed for weight loss. I have some comments to modify the manuscript.

*R#1-1:* *A paper has already been published in 2024 in which the researchers prepared the PMMA/SiO_2_-TiO_2_ composite. Is your synthetic protocol different from it? Please justify.*

Reply R#1-1: We have read the article recommended by the reviewer, which describes the synthesis method of the coating, which is different from our synthesis. In our case, it is synthesized *in situ* with sonochemical support, reducing the synthesis time to one hour, which improves the dispersion of the inorganic particles in the matrix. This process does not require curing of the hybrid material. The method of coating the substrates by blade coating, which allows to control the thickness in wet and dry in three hours, while the mentioned article is not in situ and the inorganic particles are synthesized separately by adding them to the matrix, with a synthesis time of 4 h and 10 h of aging. The coating method is by immersion and on glass substrate; the drying time is with temperature. However, this work has allowed us to know another preparation method, so we have added to our literature review. It is also noted that according to the DOI provided, the year of publication is 2004, which has been corrected in the citation.

*R#1-2: You can again check the reference style of the journal*

Reply R#1-2: The journal template was revised in detail and the article was adapted to the points required in the new version of the manuscript.

*R#1-3: How is the interaction between titanium oxide and silicon oxide identified in the Raman spectrum, and what is the wavenumber range for the Ti-O-Si bond?*

Reply R#1-3: The interaction between titanium oxide and silicon oxide is identified between 1000-1100 cm^-1^, associated with the Ti-O-Si bond, in agreement with what is reported in the literature. In our samples, the absorption band is found at 1104 cm^-1^ in sample M1 and at 1110 cm cm^-1^ in sample M2 indicating the presence of this bond.

*R#1-4: What effects does the Raman spectrum's indication of the interaction between silicon oxide and titanium oxide have on the general characteristics and possible uses of the composite material?*

Reply R#1-4: The main objective of using Raman spectroscopy is to follow the degradation of the functional groups of the material by monitoring their absorption bands before and after their evaluation under ultra-high vacuum conditions. The Ti-O-Si particles influence the mechanical properties such as adhesion and hardness, being a ceramic, so it was also monitored in the FTIR and Raman spectra. The possible applications, independent of its use this sector, could be as coating on other substrates such as glass, phenolic resin, etc., which can be used in different components of this sector.

R#1-5: *The figures' quality is very poor. Kindly improve it. As you can see in the figure of SEM and figure 13, and 14, even the digits are not visible.*

Reply R#1-5: Agreed that the figures were not of adequate quality to be observed. The SEM images and figures 13 and 14 corresponding to the Raman and FTIR spectroscopy plots have been revised to ensure that the quality and details are as clear as possible. However, each of the images has been revised to improve the resolution and quality of the figures in the revised version of the final manuscript. Thank you for your comments on the quality of the figures.

*R#1-6: What is the novelty of your research paper? Kindly discuss it briefly in the conclusion section.*

Reply R#1-6: His observation is very accurate in order to highlight the novelty of our research. In the conclusions section, we add a clearer discussion about the novelty of the work, highlighting the reduction of the synthesis time, in addition to the fact that sonochemistry is used to achieve a better homogeneity to improve its final properties. We know that this hybrid has been tested its properties in terrestrial environmental conditions for different applications; our proposal is to apply it in the aerospace sector, so we evaluate its properties in ultra-high vacuum conditions.

The final conclusion is:

The hybrids were successfully synthetized by the sol-gel in situ processing of silica-titanium particles in the presence of the PMMA matrix assisted by sonochemistry. The use of sonochemistry allowed to accelerate the initial chemical reactions, reducing the synthesis time compared to the other methods without ultrasound, as well as to have a dispersion of the Ti-O-Si particles homogeneously distributed within the polymeric matrix.

Hence, these coatings exhibit remarkable transparency due to the sonochemical synthesis, which allows homogeneity of the chemical interaction of TiO2-SiO2 (Ti-O-Si) particles and their appropriate molar composition, which is not easy to achieve with other conventional coatings. This has a significant influence on their morphological, thermal and mechanical properties.

The combination of vacuum endurance testing with Raman y FTIR spectroscopy techniques provide an in-depth and detailed evaluation of materials and components. It enables the detection of the chemical and structural changes that occur under vacuum conditions. This integrated approach is critical in the development of materials and products that must operate in low-pressure environments to ensure reliability and performance.

The prepared M1 and M2 coatings provide a reliable option for the aerospace industry because of the importance of the ultra-high vacuum resistance of the coatings on Al 7075 substrates. After the high-vacuum test, their adhesion was significantly good, showing a peel percentage of no more than 5 % and an average hardness of 4H, their weight loss was no more than 0,02 %, which meets the established standard.

In general, the synthesized coatings did not show any significant chemical or mechanical degradation, which allows us to propose them as coatings in the aerospace sector.

Further studies of these hybrid materials we are related to other evaluations for a passive thermal control system and for analyzing their resistance to low temperatures. As well as their evaluation for other environmental conditions such as resistance to atomic oxygen attack, radiation resistance and to the effects of cosmic dust. The preparation these coatings would open a gap for their applications on other industrial sectors.

**Reviewer #2:**

The article "Hybrid Coating Prepared with PMMA/Ti-O-Si Tested Under Vacuum Conditions for Use in Nanosatellites" investigates the development and performance of a PMMA/SiO_2_-TiO_2_ hybrid coating applied to Al 7075 alloys, crucial in aerospace applications. The hybrid coatings, synthesized via sol-gel reaction assisted with sonochemistry, integrate SiO_2_ and TiO_2_ particles (rutile/anatase mix) via TEOS and TIPO precursors. Radical polymerization of MMA monomer with 3-MPTS coupling agent and benzoyl peroxide catalyst ensures a homogeneous, defect-free coating with strong adhesion and a hardness of 4 H, approximately 20 µm thick. Addressing the bellow questions would strengthen the article's impact and broaden its applicability in advanced material sciences.

*R#2-1: The article claims minimal weight loss (0.02%) and enhanced chemical stability under vacuum (10^-5^ Torr, 125 °C). How was this stability quantitatively measured, and were there comparative studies with other coatings or conditions?*

Reply R#2-1: The chemical stability was determined by FTIR and Raman spectroscopy, according to the presence of absorption bands in the spectra of chemical bonds of the functional groups -Ti-O-Si-, -C=O and –OH of the coating on the substrate before and after the vacuum test, and no shift of the bands is observed. These bands provide us with the adhesion, hardness and abrasion, since these were not significantly modified at the end of the vacuum resistance test, it is inferred that the chemical stability of the material is adequate.

On the other hand, the synthesized hybrid we have not compared it with other commercial coatings, but other ah doc articles have been reviewed, but their mechanical properties are evaluated under terrestrial environmental conditions and not to space environment conditions as our research. However, other hybrid coatings with different polymer matrices and inorganic particles have been reported, evaluating their resistance in ultra-high vacuum conditions.

Reference:

<https://doi.org/10.1016/j.vacuum.2021.110228>

<https://doi.org/10.1016/j.matchemphys.2008.07.036>

*R#2-2: Authors need to edit their research work so that it is clearer what the study's topic and methodology are. Authors may cite, for example, literature outlining the interest in recent study results released by researchers to bolster their introduction part.*

[https://doi.org/10.3390/pr11030959](https://doi.org/10.3390/pr11030959), [https://doi.org/10.1016/j.surfcoat.2024.131128](https://doi.org/10.1016/j.surfcoat.2024.131128), [https://doi.org/10.1016/j.scriptamat.2023.115763](https://doi.org/10.1016/j.scriptamat.2023.115763), [https://doi.org/10.1016/j.jmst.2023.05.082](https://doi.org/10.1016/j.jmst.2023.05.082).

Reply R#2-2: The proposed section has been properly edited, we certainly made a mistake and left the abstract as follows:

“A hybrid coating made of poly (methyl methacrylate) with SiO_2_-TiO_2_ particles (PMMA/SiO_2_-TiO_2_) has been developed for use as a coating on nanosatellites, evaluating its resistance to high vacuum by quantifying its weight loss. The coating was applied on an Al 7075 aluminum substrate used for the aerospace sector. PMMA/SiO_2_-TiO_2_ hybrid coatings were prepared using sol-gel reaction in situ assisted with sonochemistry. The SiO_2_ particles and TiO_2_ (50:50 % wt. of rutile/anatase) particles by tetraethyl orthosilicate (TEOS), and Titanium (IV) Isopropoxide (TIPO). Radical polymerization of methyl methacrylate (MMA) monomer was conducted with (3-mercaptopropyl) trimethoxy silane (3-MPTS) used as a coupling molecular agent, and benzoyl peroxide as a catalyst. The coatings obtained have a thickness of 20 µm which were deposited by blade coating technique on the substrate, obtaining homogeneous and defect-free coatings. Adhesion and hardness were measured using ASTM standards required for this sector. To evaluate its resistance to ultra-high vacuum, it was done as close as possible to the ASTM E-595 norm, where it indicates that the coatings must be evaluated at vacuum conditions of 10-5 Torr and 125 °C for a period of 24 hours. The coatings were evaluated before and after the test by spectroscopy analysis to determine a possible degradation in the chemical structure. The resulting weight loss not exceeding 0.02 %, and the addition of Ti-O-Si particles led to an increase in chemical stability under vacuum conditions without affecting the chemical structure of the highly cross-linked PMMA/Ti-O-Si matrix, which was monitored by FTIR and Raman spectroscopy.”

The objective of this work was to synthesize a hybrid coating by sonochemical assisted sol-gel process for its application in Al 7075 nanosatellites. Its chemical and mechanical properties have been determined before and after its evaluation in high vacuum resistance. A bibliographic review has been made as recent as possible, maximum 5 years ago, and indeed current references have been found among them two of the four proposed, they have been carefully read and have been incorporated to the introduction, which has strengthened our research.

*R#2-3: FTIR and Raman spectroscopy were used to monitor the chemical structure before and after vacuum testing. Could the authors elaborate on specific changes observed in the spectra and correlate them with the coating's performance metrics?*

Reply R#2-3: The characteristic absorption bands of the chemical structure of the coating were monitored by FTIR and Raman spectroscopy, mainly looking for not shift or disappearance of the functional groups, which would indicate a degradation of the material affecting the mechanical performance of the coating. In addition, the intensities of the characteristic bands of the functional groups -Ti-O-Si-, -C=O and –OH were analyzed in both Raman and FTIR and no significant difference was observed. The plots have been edited to better visualize these changes.

R#2-4: *Has the durability of the PMMA/SiO_2_-TiO_2_ coating been assessed over extended periods or under cyclic vacuum conditions that mimic operational scenarios for nanosatellites?*

Reply R#2-4: Yes, it is definitely the goal of the work to adhere as closely as possible to ASTME 595, “Standard Test Method for Total Mass Loss and Collected Volatile Condensable Materials from Outgassing in a Vacuum Environment”, which is used for organic, polymeric, and inorganic materials.

The standard states: “Which indicates that while the 24-hour test time does not represent actual outgassing from years of operation, the standard specifies the higher test temperature and shorter time to allow comparisons of materials without the intent of predicting actual outgassing in service. The 125 °C test temperature was assumed to be significantly higher than the expected service temperature. If expected service temperatures exceed 65 to 70 °C, the test temperature should be increased. It is recommended that the test temperature be at least 30 °C higher than the maximum expected service temperature to provide material comparisons for TML”

For the mechanical tests, the ASTM D 3359-17 and ASTM D3363 standards were used, with reference to what is stated in the technical data sheets of the commercial products. However, testing of commercial products was not compared to our hybrid.

R#2-5: *What are the challenges and considerations in scaling up the synthesis of these coatings for industrial applications, particularly in the context of large-scale production for aerospace industries?*

Reply R#2-5: This coating was designed to be used as a passive thermal control system in the interior of the nanosatellites, which are of the order of 10 x 10 x 10 x 10 cm. On an industrial level, it would only be a matter of scaling up the quantities of reagents and exploring different deposition methods, depending on the size of the substrate.

The limiting factor would be the volume of material to be synthesized, since the sonotrode would have to be adequate for that volume.

R#2-6: *Considering the promising results, what are the next steps in research or potential modifications to further enhance the performance or broaden the application scope of PMMA/SiO2-TiO2 coatings?*

Reply R#2-6: The authors are grateful for this comment, because it is very gratifying to know that other colleagues agree that the research is very promising, since the proposal of the work is the synthesis of the material so that it can be used as a coating.

To improve our research, we will continue with the evaluation of the coating as a passive thermal control system and analyze its resistance to low temperatures. We will also evaluate it to other environmental conditions such as resistance to atomic oxygen attack, radiation resistance as well as evaluate its resistance to the effects of cosmic dust.

On this basis, there are additional challenges in the application of theses materials to other substrates in order to expand their potential applications to other industrial sectors.

R#2-7: *The authors should compare their results with previous studies, which are included in a table of references below.*

*[https://doi.org/10.1016/j.corsci.2023.111591](https://doi.org/10.1016/j.corsci.2023.111591),*

[*https://doi.org/10.1007/s40194-024-01704-w*](https://doi.org/10.1007/s40194-024-01704-w)*,*

[*https://doi.org/0.1109/TNSE.2023.3342938*](https://doi.org/0.1109/TNSE.2023.3342938)

[*https://doi.org/10.1002/sus2.228*](https://doi.org/10.1002/sus2.228)

Reply R#2-7: The proposed articles give us a wide range of applications, but we cannot specifically compare them with the synthesized material, since we are not proposing it for the aeronautical sector. However, in parallel with the work, we are investigating these possible applications in this sector, but without doubt these previous works will open future expectations in our working group.

The reference: <https://doi.org/0.1109/TNSE.2023.3342938>, it was not possible for us to verify it because it allowed us to open it.
